# Remission einer therapierefraktären generalisierten pustulösen Psoriasis unter dem Interleukin-36-Rezeptor-Inhibitor Spesolimab

**DOI:** 10.1007/s00105-023-05140-7

**Published:** 2023-03-21

**Authors:** Valentina Laura Müller, Alexander Kreuter

**Affiliations:** 1 Klinik für Dermatologie, Venerologie und Allergologie, Helios Klinikum Duisburg, Duisburg, Deutschland; 2 Klinik für Dermatologie, Venerologie und Allergologie, Klinik für Dermatologie, Venerologie und Allergologie, HELIOS St. Elisabeth Klinik Oberhausen, Universität Witten/Herdecke, Josefstr. 3, 46045 Oberhausen, Deutschland

**Keywords:** Psoriasis vulgaris, Sterile Pustelbildung, Inflammation, Erythematöse Haut, Krankheitsaktivität, Psoriasis vulgaris, Sterile pustule formation, Inflammation, Erythema, Skin diseases

## Abstract

Die generalisierte pustulöse Psoriasis (GPP) ist eine inflammatorische Erkrankung, die prinzipiell lebensbedrohlich verlaufen kann. Im Gegensatz zur „klassischen“ Psoriasis (Psoriasis vulgaris) geht diese mit einer sterilen Pustelbildung auf erythematöser Haut einher. Bislang gab es in Europa keine suffiziente, zugelassene Therapieoption, sodass die für die Psoriasis vulgaris eingesetzten Medikamente auch bei der GPP Verwendung fanden. Neuere Studien belegen, dass bei der GPP oft eine Mutation des Interleukin-36-Rezeptor-Antagonisten (IL-36Ra) zu einer gesteigerten Inflammation und entsprechend zur Krankheitsaktivität führt. Wir berichten den Fall einer schweren GPP mit einer kompletten Remission nach 2‑maliger Gabe von Spesolimab, einem neuen Interleukin-36-Rezeptor-Antikörper.

## Abstract (en)

Generalized pustular psoriasis (GPP) is often a life-threatening disease which, in contrast to psoriasis vulgaris, is associated with the formation of sterile pustules on the skin. Until recently, there was no approved treatment in Europe, so that drugs used for psoriasis vulgaris were also frequently used for GPP. However, new studies showed that, in GPP, mutation of the interleukin-36 receptor often leads to increased inflammation and corresponding disease activity. We report a case of GPP with complete remission after two doses of spesolimab, a new interleukin-36 receptor antibody.

## Anamnese

Ein damals 63-jähriger Mann stellte sich im August 2018 erstmalig in unserer Notaufnahme mit generalisierter Pustelbildung der Haut, subfebriler Temperatur (38,2 °C), Tachykardie (160/min), Hypertonie (160/100 mm Hg) und deutlicher Allgemeinzustandsverschlechterung vor. Die Hautveränderungen seien 4 Tage zuvor akut aufgetreten und hätten sich innerhalb kürzester Zeit auf den gesamten Körper ausgebreitet. Mehrere Wochen zuvor habe er unter abdominellen Beschwerden gelitten, die im Rahmen einer Koloskopie als Colitis ulcerosa diagnostiziert und mit Prednisolon und Mesalazin behandelt worden seien.

## Untersuchung

**Figure 1:**
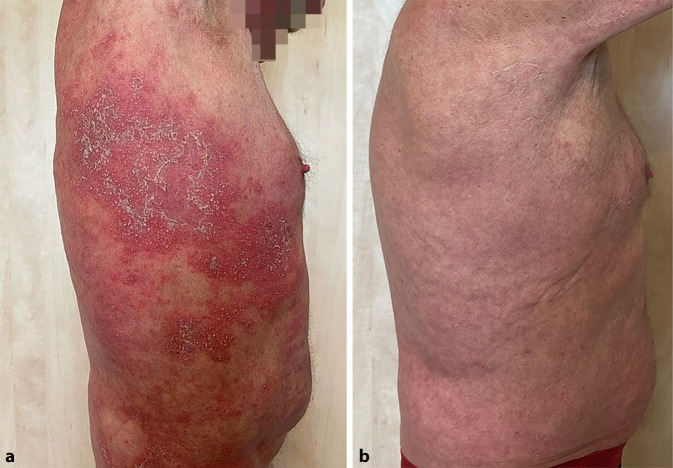

Im Rahmen der Erstvorstellung war nahezu das gesamte Integument von großflächig konfluierenden, fragilen Pusteln bedeckt (Abb. 1a und 2a). Des Weiteren zeigten sich ein Exsikkationsekzem sowie mild ausgeprägte Unterschenkelödeme. Die Schleimhäute waren trocken, die Lymphknoten nicht vergrößert palpabel. In der orientierenden körperlichen Untersuchung wies der Patient keine weiteren Pathologika auf.

**Figure 2:**
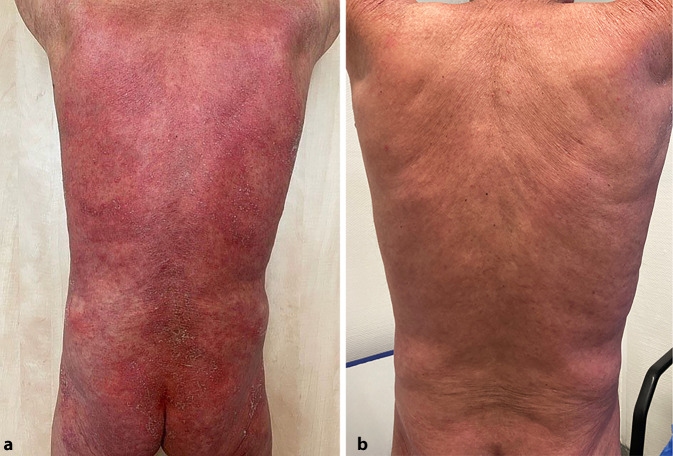

## Diagnostik

Im Labor zeigten sich eine Leukozytose (24,88/nl), Neutrophilie (94,4 %) sowie ein erhöhtes CRP (C-reaktives Protein) (25,65 mg/dl) und Procalcitonin (1,18 ng/ml). Die mikrobiellen Abstriche aus dem Pustelinhalt ergaben keinen Nachweis für pathologische Keime. Histopathologisch zeigte sich neben einem akanthotischen, leicht abgeflachten Deckepithel eine ausgeprägte Spongiose sowie eine intra- und subkorneale Ansammlung neutrophiler Granulozyten. Daneben imponierten eine ödematöse Auflockerung des Koriums sowie ein diffuses Entzündungsinfiltrat aus Lymphozyten, Histiozyten und neutrophilen Granulozyten. In Zusammenschau der klinischen und histopathologischen Befunde stellten wir die Diagnose einer generalisierten pustulösen Psoriasis (GPP).

## Therapie und Verlauf

Nach Diagnosestellung einer GPP erfolgte zur akuten Stabilisierung des Patienten zunächst eine hoch dosierte Prednisolon-Therapie intravenös mit 100 mg täglich, worunter die Pustelbildung sistierte. Bei jedoch weiterhin bestehender Suberythrodermie und erneutem Aufflammen der Läsionen bei niedrigeren Prednisolon-Dosen entschlossen wir uns zur Initiierung einer Therapie mit Methotrexat (MTX) 15 mg subkutan pro Woche. Topisch kam Mometasonfuroat-Salbe 1‑mal täglich zur Anwendung. Zunächst besserte sich der Hautbefund schrittweise in den folgenden Wochen, bis dann kurze Zeit später ein erneuter Schub auftrat, sodass die Therapie im November 2018 um Infliximab 5 mg/kg Körpergewicht als intravenöse Gabe zusätzlich eskaliert wurde. Im Rahmen der Behandlung kam es jedoch 2‑malig zu Episoden einer Sepsis, die eine stationäre Aufnahme zur intravenösen Antibiotikatherapie notwendig machten. Die Kombinationstherapie aus MTX und Infliximab wurde bis September 2021 fortgeführt. Trotz hochgradig immunsuppressiver Therapie kam es zu regelmäßigen Rezidiven, sodass die Schübe intermittierend mit sowohl oralem, als auch intravenösem Prednisolon abgefangen werden mussten. Eine Entwöhnung vom topischen Mometasonfuroat konnte zu keinem Zeitpunkt erfolgen. Im September 2021 wurde letztlich aufgrund einer Schubzunahme, anamnestisch durch Impfungen mit dem SARS-CoV-2-Impfstoff BNT162b2 getriggert, die bestehende Medikation mit MTX und Infliximab abgesetzt und auf den Interleukin-23-Inhibitor Risankizumab umgestellt. Auch unter der neuen Medikation schien sich der Hautbefund zunächst zu stabilisieren, verblieb jedoch subklinisch mit insbesondere an den Flanken persistierenden Pusteln, gelegentlich auftretender Suberythrodermie sowie schubweisem Aufflammen generalisierter Pusteln. Eine Erscheinungsfreiheit war zu keinem Zeitpunkt mehr zu erreichen. Im Mai 2022 wurde auch diese Therapie beendet.

Es erfolgte daraufhin mit Absetzen von Risankizumab die probatorische Gabe des Interleukin-36-Rezeptor-Antikörpers Spesolimab. Da sich das Medikament zu diesem Zeitpunkt noch in der Zulassung befand, konnte die Applikation im Rahmen des „compassionate use programs“ durchgeführt werden. Der Patient erhielt im akuten Schub 900 mg Spesolimab per infusionem über insgesamt 90 min an Tag 0, worunter es nach etwa 3 Tagen zu einem deutlichen Rückgang des massiven Juckreizes und der Pustelbildung kam. Da nach 1 Woche noch keine gänzliche Erscheinungsfreiheit zu erzielen war, gaben wir nach Protokoll des Herstellers erneut 900 mg Spesolimab [1]. Hierunter kam es zu einem vollständigen Sistieren der Pustelbildung und einem deutlichen Rückgang der Erytheme. Erstmalig konnte die topische, steroidale Therapie abgesetzt werden, ohne dass es zu einem Rückfall kam. Bis zum jetzigen Zeitpunkt präsentiert der Patient einen insgesamt zufriedenstellenden Hautbefund und erlitt kein erneutes Rezidiv (Abb. 1b und 2b). Auch die Colitis ulcerosa blieb im gesamten Behandlungszeitraum klinisch inapparent. Die Therapie wurde bislang ohne weitere Nebenwirkungen oder Beschwerden vertragen.

## Diskussion

Die GPP ist eine entzündliche, zum Teil lebensbedrohliche Erkrankung, die oft schubweise verläuft und innerhalb weniger Stunden zu einer generalisierten Pustelbildung des gesamten Körpers führen kann. Oftmals werden die Schübe von Fieber und Gliederschmerzen begleitet, häufig finden sich eine Leukozytose und CRP-Erhöhung [2]. Bislang wurde die GPP zum Formenkreis der Psoriasis gezählt und tritt gehäuft auch gleichzeitig mit dieser auf. Neuere Studien belegen allerdings einen unterschiedlichen Pathomechanismus für beide Erkrankungen, sodass die GPP mittlerweile als eigene Entität gilt [3]. Die Sepsis ist eine gefürchtete Komplikation im akuten Schub [4] und führte auch bei unserem Patienten 2‑malig zur Hospitalisierung.

Als teilweise ursächlich für die GPP wird eine Mutation des Interleukin-36-Rezeptor-Antagonisten (IL-36Ra) postuliert [5]. Insgesamt sind 4 Interleukin-36-Zytokine bekannt, von denen 3 proinflammatorisch wirken (IL-36alpha, IL-36beta und IL-36gamma) und eines antiinflammatorisch agiert (IL-36Ra). Die genannten Zytokine dienen der antimikrobiellen Abwehr und wirken agonistisch am Interleukin-36-Rezeptor (IL-36R). Eine Mutation des inhibitorisch wirkenden IL-36Ra im Sinne einer „loss of function mutation“ resultiert daher in einer gesteigerten Inflammation [6].

In Europa und den USA gab es bis vor Kurzem keine zugelassenen Behandlungsoptionen mit Biologika, während in Japan diverse Therapien zur Verfügung stehen, die üblicherweise bei der Psoriasis vulgaris eingesetzt werden (u. a TNF[Tumornekrosefaktor]-α‑, IL-17- und IL-23-Inhibitoren). Die Zulassungsstudien fußen jedoch – unter anderem aufgrund der Seltenheit der Erkrankung – zumeist auf sehr kleinen Fallzahlen, einarmigen, offenen Studien sowie Fallberichten [7].

Der humanisierte, monoklonale Ig(Immunglobulin)G1-Antikörper Spesolimab ist eine der ersten Substanzen, die spezifisch den IL-36-Rezeptor inhibieren und zu einer Reduktion der proinflammatorischen Kaskade führen. In einer Phase-2-Studie konnte gezeigt werden, dass die Gabe von Spesolimab zu einem deutlichen Rückgang der Hautläsionen führte. Primärer Endpunkt war in dieser Studie der Rückgang der Pusteln, der mittels Generalized Pustular Psoriasis Physician Global Assessment (GPPGA) pustulation subscore dokumentiert wurde. Von insgesamt 53 eingeschlossenen Patienten (35 im Spesolimab-Arm und 18 im Placeboarm) zeigte sich bereits 1 Woche nach der Spesolimab-Infusion bei 19 (54 %) Fällen im Verumarm ein GPPGA von 0, während dies nur bei 1 (6 %) Fall unter Placebo eintrat. Nebenwirkungen wie eine erhöhte Infektionsgefahr traten unter Therapie häufiger auf als unter Placebo [8]. Interessanterweise scheint Spesolimab auch bei fehlender Mutation des IL-36Ra (Mutation im *IL-36RN*-Gen) wirksam zu sein [9]. Seit Januar 2023 ist Spesolimab nun auch auf dem Markt verfügbar und für den akuten Schub als Monotherapie zugelassen.

Weitere Therapien, wie z. B Imsidolimab, die den Fokus auf IL-36-Zytokine oder deren Rezeptor legen, befinden sich in der Erprobung und scheinen ebenfalls eine gute Wirksamkeit in der Therapie der GPP zu erzielen [10].

## Fazit für die Praxis

- Die generalisierte pustulöse Psoriasis (GPP) ist eine seltene, potenziell lebensbedrohliche Erkrankung.
- Aufgrund des unterschiedlichen Pathomechanismus wird die GPP als eigene Entität unabhängig von der „klassischen Psoriasis“ (Psoriasis vulgaris) angesehen.
- Mit den neuen Interleukin-36-Inhibitoren gibt es nun erstmalig vielversprechende Therapieoptionen, die gezielt in den Pathomechanismus der Erkrankung eingreifen.
